# Supplementary material for: Plant-Programmed Cell Death-Associated Genes Participation in Pinus sylvestris L. Trunk Tissue Formation
Source: Plants (Basel). 2022 Dec 9;11(24):3438. doi: 10.3390/plants11243438 (PMC9785643; doi:10.3390/plants11243438)
Supplement: Supplementary file 1 [file plants-11-03438-s001.zip › plants-2034642-supplementary.pdf]

plants

MDPI

Supplementary Material

Plant Programmed Cell Death Associated Genes Participation in *Pinus sylvestris* L. Trunk Tissues Formation

Moshchenskaya Yu.L.[1]*, Galibina N.A.[1], Nikerova K.M.[1], Tarelkina T.V.[1], Korzhenevsky M.A.[1], Sofronova I.N.[1], Ershova M.A.[1], Semenova L.I.[1]

Forest Research Institute, Karelian Research Centre of the Russian Academy of Sciences, 11 Pushkinskayast., Petrozavodsk, Russia, 185910; tselishcheva.yulia@mail.ru (Y.L.M.); galibina@krc.karelia.ru (N.A.G.); ershova.maria@krc.karelia.ru(M.A.E); knikerova@yandex.ru (K.M.N.); karelina.t.v@gmail.com (T.V.T.); mkorz@krc.karelia.ru (M.A.K.); irinasof@krc.karelia.ru (I.N.S.); mi7enova@gmail.com (L.I.S.).

* Correspondence: tselishcheva.yulia@mail.ru; Tel.: +7(8142)768160

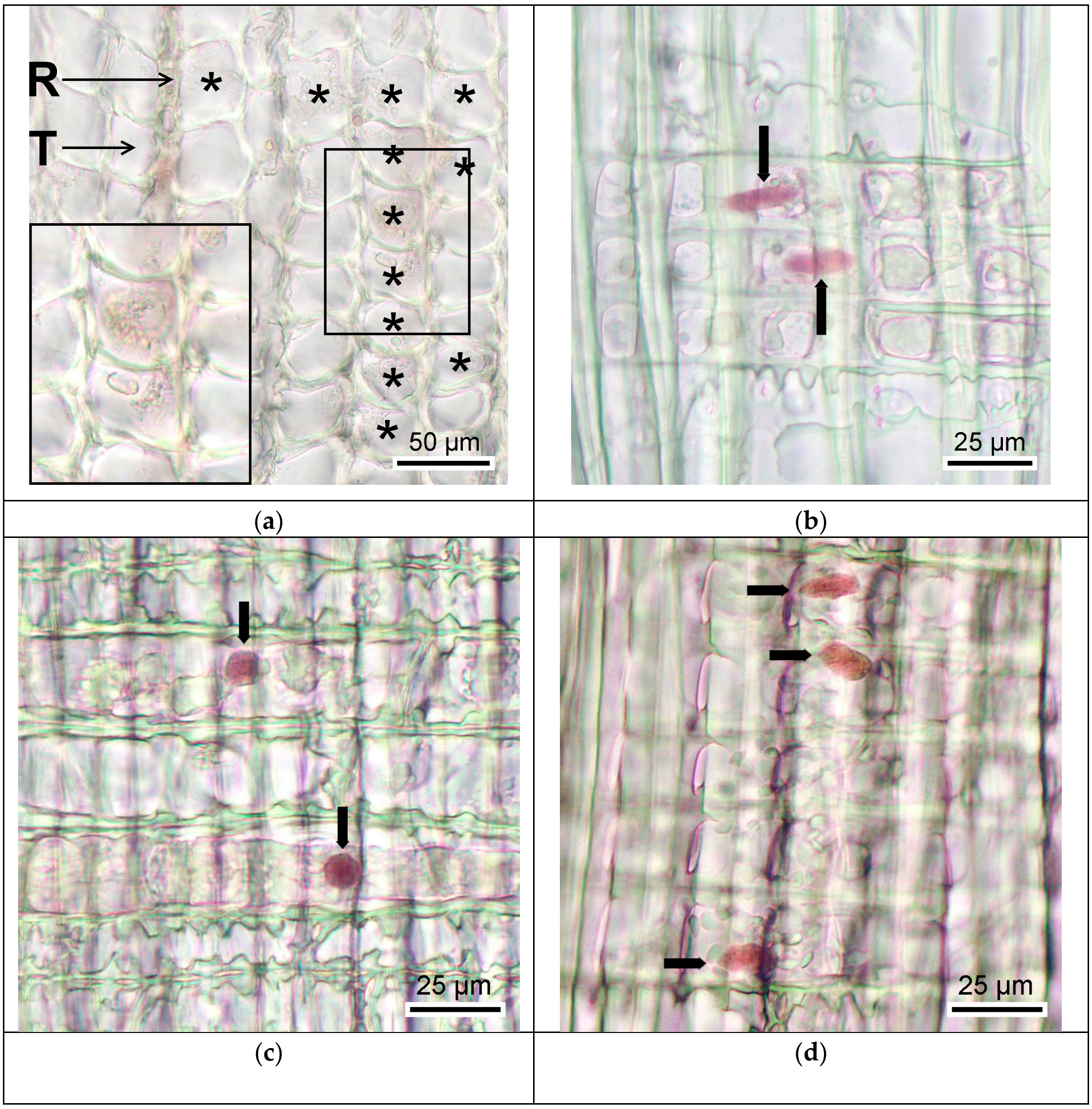

(**a**) (**b**)

(**c**) (**d**)

Supplementary Figure S1. (**a**) Transverse section of differentiating xylem (Fraction 2). R - ray, T - tracheid. The cellular contents in differentiating tracheids are visible (shown with asterisks). In the inset, two cells are shown at higher magnification. (**b**) Radial section of differentiating xylem (Fraction 2). The nuclei in the ray parenchyma cells are oblong-elliptical (black arrows). (**c**) Radial section of inner sapwood (SWin). The nuclei in the ray parenchyma cells display evident pycnotic

degeneration. They are rounded due to water loss and chromatin condensation (black arrows). (**d**) Radial section of transition zone (TZ). The destruction of the nuclear membranes occurs. Chromatin stained with acetocarmine can be observed in cells (black arrows).
